# Medical Society of Georgia

**Published:** 1861-03

**Authors:** 


### EDITORIAL AND MISCELLANEOUS.

### Medical Society of Georgia.

It will be remembered that the Medical Society of the State will convene in Atlanta on the second Wednesday in April, proximo. We hope the time will be kept in mind by physicians throughout the State, so that a large number may be in attendance. In addition to the usual reports and discussions on medical subjects, business of importance, connected with the profession of the State, will be transacted at the approaching session.

These annual reunions of members of the medical profession afford the means of mutual benefit to each other as practical physicians, and an amount of satisfaction in the greeting of professional brethren sufficient amply to reward all who attend the deliberations of the body. Come one, come all!

We hope other journals in the State will call attention to the time and place of meeting, so that we may have a meeting worthy the Empire State of the Confederacy, and worthy the noble cause of practical medicine.

